# Oral or parenteral administration of curcumin does not prevent the growth of high-risk t(4;11) acute lymphoblastic leukemia cells engrafted into a NOD/SCID mouse model

**DOI:** 10.3892/ijo.2012.1734

**Published:** 2012-12-12

**Authors:** SUSAN J. ZUNINO, DAVID H. STORMS, JOHN W. NEWMAN, THERESA L. PEDERSEN, CARL L. KEEN, JONATHAN M. DUCORE

**Affiliations:** 1 United States Department of Agriculture, Agricultural Research Service, Western Human Nutrition Research Center; Davis, CA 95616;; 2 Department of Nutrition, University of California Davis, Davis, CA 95616;; 3 Department of Pediatrics, Section of Hematology/Oncology, University of California School of Medicine, Sacramento, CA 95817, USA

**Keywords:** acute lymphoblastic leukemia, curcumin, non-obese diabetic/severe combined immunodeficient mice

## Abstract

In this study, the efficacy of orally and parenter-ally administered curcumin was evaluated in non-obese diabetic/severe combined immunodeficient (NOD/SCID) mice (NOD.CB17-Prkdc^scid^/J mice) engrafted with the human t(4;11) acute lymphoblastic leukemia line, SEM. SEM cells were injected into the tail vein and engraftment was monitored by flow cytometry. Once engraftment was observed, the chemotherapeutic potential was examined by injecting mice intraperitoneally with curcumin (5 mg/kg body weight) dissolved in dimethylsulfoxide (DMSO) or DMSO alone (control) every other day, or vincristine (0.5 mg/kg body weight) 3 times per week for 4 weeks (n=16 per group). The intraperitoneal administration of curcumin did not inhibit the growth of the leukemia cells. To determine the efficacy of oral curcumin, mice were fed a control diet or a diet containing 0.5% w/w curcumin 3 weeks prior to the injection of the leukemia cells and throughout the experimental period (n=16 per group). To determine whether dietary curcumin can enhance the efficacy of a conventional chemotherapeutic agent, vincristine was injected intraperitoneally into leukemic mice fed the different diets. Dietary curcumin did not delay the engraftment or growth of leukemia cells, or sensitize the cells to vincristine. Liquid chromatography-tandem mass spectrometry analyses of mouse sera showed that curcumin rapidly metabolized to glucuronidated and sulfated forms within 1 h post-injection and these were the major curcumin metabolites found in the sera of the mice fed the curcumin diet. In contrast to the findings in previous *in vitro* models, the current data indicate that orally or parenterally administered curcumin is not a potent preventive agent against high-risk t(4;11) acute lymphoblastic leukemia.

## Introduction

Curcumin [1,7-*bis*(4-hydroxy-3-methoxyphenyl)-1E,6E-heptadiene-3,5-dione] is a bioactive polyphenol found in turmeric (*Curcuma longa*). Curcumin has been reported to inhibit the activity of a variety of signaling enzymes in cells, such as NF-κB, mitogen-activated protein kinases, cyclooxygenase-1, Bcl-2, Bcl-xL and cyclin D1, that contribute to cellular survival and proliferation (reviewed in refs. 1–3). Given the above effects of curcumin, it has been widely touted as a potential anti-carcinogenic agent and its efficacy has been investigated in numerous clinical trials (reviewed in ref. 3). While the interest in curcumin as a potential anti-carcinogenic agent is understandable, it is important to note that many of the putative anti-cancer activities of this phytochemical are based on data obtained from *in vitro* studies using supra-physiological concentrations of the parent compound. However, *in vivo,* curcumin undergoes rapid and extensive metabolic transformations that largely results in circulating glucuronidated and sulfated curcumin or tetrahydro- and hexahydrocurcumin metabolites that have poorly documented functions (4,5). Therefore, the physiological significance of the *in vitro* investigations in which the parent compound was used may be questioned. With the above noted, several animal studies have suggested a potential role for curcumin in the prevention of select cancers. Curcumin has been reported to effectively suppress tumor initiation by benzo[a]pyrene and 7,12-dimethyl-benz[a]anthracene and inhibit tumor promotion induced by phorbol ester in mice (6,7). Similarly, dietary curcumin has been reported to have potent chemopreventive activity by suppressing the development of stomach and colon cancers (8), radiation-induced mammary tumors (9,10) and hepatocarcinogenesis (11) in animal models. Dietary curcumin has been reported to inhibit the metastasis of human breast cancer to the lung in immunodeficient mice (12,13). Curcumin administered orally at 100 and 20 mg/kg body weight has been reported to increase the survival of Balb/c mice with erythroleukemia induced by infection with Friend murine leukemia virus and to decrease the leukemia cell infiltration of the spleen (14). Daily intraperitoneal (i.p.) injections of curcumin at a concentration of 40 mg/kg body weight have been shown to lead to an increase in the survival of Balb/c mice engrafted with B-acute lymphoblastic leukemia, although survival was only increased by 11 days compared to the control mice (15).

In the current study, we investigated the *in vivo* efficacy of orally and parenterally administered curcumin against a high-risk acute lymphoblastic leukemia (ALL) cell line with the chromosomal translocation t(4;11). The t(4;11) translocation is found in 60–80% of infants with ALL and this high-risk leukemia has a poor prognosis (16,17). The SEM line was established from a relapsed patient with t(4;11) ALL (18) and used in our experiments to engraft non-obese diabetic/severe combined immunodeficient (NOD/SCID) mice. The ability of dietary or i.p. administered curcumin to reduce or prevent leukemia cell growth, sensitize the leukemia cells to the chemotherapeutic agent, vincristine, or delay engraftment in these mice was evaluated. Serum concentrations of curcumin, curcumin glucuronide and curcumin sulfate were analyzed in a subset of mice orally or i.p. administered curcumin to determine whether sufficient levels of curcumin were present to prevent leukemia cell growth in this mouse model.

## Materials and methods

### Cells and reagents

The SEM cell line containing the t(4;11) translocation (18) was grown in RPMI-1640 (Invitrogen Life Technologies, Carlsbad, CA) supplemented with 10% fetal bovine serum (Sigma-Aldrich, St. Louis, MO), 1 mmol/l sodium pyruvate, 2 mmol/l L-glutamine, 50 IU/ml penicillin, 50 *μ*g/ml streptomycin, 0.25 *μ*g/ml amphotericin B (Invitrogen Life Technologies) and incubated at 37°C with 5% CO_2_. SEM cells were washed twice in Dulbecco’s phosphate-buffered saline (PBS) without Ca^2+^ or Mg^+^ (Sigma-Aldrich) and resuspended in PBS at a final concentration of 50x10^6^ cells/ml before injection.

Vincristine sulfate was purchased from Sigma-Aldrich and dissolved in PBS. For injection studies, curcumin (>98% pure) was purchased from Axxora LLC (San Diego, CA) and dissolved in dimethylsulfoxide (DMSO; Sigma-Aldrich). The solutions were filter-sterilized, aliquoted and frozen at −20°C until use. For the feeding experiment, curcumin (>90% pure) was purchased in bulk from Cayman Chemical Co. (Ann Arbor, MI). Phycoerythrin-cyanine 7 (PE-Cy7)-conjugated anti-human CD19 and allophycocyanin-Cy7 (APC-Cy7)-conjugated anti-mouse CD45 antibodies were purchased from Becton-Dickinson (San Jose, CA). For mass spectrometric (MS) analyses, the following reagents were purchased: dimethoxycurcumin and 1-cyclohexyluriedo-3-dodecanoic acid (CUDA) (Cayman Chemical Co.), sulfatase from *Aerobacter aerogenes*, β-glucuronidase (Type IX-A) from *Escherichia coli*, formic acid, glycerol, potassium 4-nitrophenyl sulfate and 4-nitrophenyl β-D-glucuronide (Sigma-Aldrich), ammonium hydroxide, LC-MS grades of methanol, acetonitrile and water (Fisher Scientific Co. LLC, Fair Lawn, NJ). Normal mouse serum was obtained from United States Biological (Swampscott, MA).

### Mice

All experimental procedures using mice were approved by the University of California Davis Institutional Animal Care and Use Committee. Female NOD.CB17-Prkdc^scid^/J mice (5–6 weeks old) were purchased from Jackson Laboratory (Bar Harbor, ME; common name, NOD/SCID mice) and housed in ventilator racks under pathogen-free conditions at the University of California, Davis *Vivarium*. The mice were maintained in a temperature-controlled environment with a 12-h light-dark cycle and provided with sterilized food and water *ad libitum*. The addition of sterile food and water and cage changes were performed in a laminar flow change-out cabinet. Mice were weighed once per week. The weighing of mice and injections of leukemia cells or chemotherapeutic agents were performed in a biosafety cabinet to reduce the risk of exposure of the animals to pathogens. Mice were euthanized by carbon dioxide asphyxiation when they became ill or at the end of the experimental period.

### Diets

Rodent Diet 7013 (Harlan Teklad, Madison, WI), a commercial NIH-31 modified diet, was used as the base diet for all experiments, as it is similar in composition to the diet used by Jackson Laboratory for the maintenance of NOD. CB17-Prkdc^scid^/J mice. For the feeding experiment, 2 intervention diets were prepared by Harlan Teklad. Diet 1 was the base diet (control) and diet 2 was the base diet containing 0.5% w/w curcumin. A dietary concentration of 0.5% w/w curcumin is equivalent to approximately 750 mg/kg body weight/day assuming a 20-g mouse and consumption of 3 g of food/day. The food was γ-irradiated for sterilization purposes. The pelleted food was packaged in 2-kg vacuum sealed bags to reduce exposure to air. Diets were stored at −20°C until use and the mice were provided with fresh food weekly.

### Detection of engraftment of the leukemia cells

At the age of 8 weeks, each mouse was injected with 5x10^6^ SEM cells through the tail vein using a 1 cc syringe with a 30 gauge needle (Becton-Dickinson). The injection volume was 100 *μ*l. The engraftment of the leukemia cells was monitored by the detection of human leukemic cells in the blood using a FACSCanto™ fluorescence-activated cell sorter (FACS) and FACSDiva™ software (Becton-Dickinson), as described previously (19). A total of 30,000 events were collected for each sample. Positive engraftment was established when the proportion of human CD19^+^ cells reached 1% in the murine peripheral blood leukocyte (PBL) population (20,21).

### Parenteral administration of curcumin

For i.p. injection, an initial dose of 100 mg curcumin/kg body weight was used as this dose has been reported to be well-tolerated in rats (22). However, this i.p. dose increased the rate of death in the leukemic mice compared to the leukemic control group. Therefore, a dose response analysis was performed using 5, 10, 25 and 50 mg curcumin/kg body weight to determine the appropriate concentration to use in our subsequent experiments (n=4 mice per group). A dose of 5 mg curcumin/kg body weight was determined to be a safe dose in these mice. Mice (8 weeks old) were injected with SEM leukemia cells as described above and randomly separated into the control, curcumin, or vincristine treatment groups (n=16 per group). Once engraftment of the leukemia cells was established, the mice were injected i.p. every other day with DMSO or curcumin (5 mg/kg body weight), or 3 times per week with vincristine (0.5 mg/kg body weight) for a total of 4 weeks. The volumes of DMSO and curcumin were 40–60 *μ*l per mouse and 80–130 *μ*l of vincristine per mouse. Body weights were measured weekly and the volumes of DMSO and therapeutic agents were adjusted accordingly. Blood from each mouse was monitored for the percentage of human CD19^+^ leukemia cells by flow cytometry. The mice were monitored daily for signs of overt illness.

### Dietary intervention with curcumin

To determine whether dietary curcumin delays the engraftment and growth of leukemia cells, 32 5-week-old mice were randomly divided into groups and fed the control diet or the diet containing 0.5% w/w curcumin (n=16 per group). After 3 weeks on the control or curcumin diet, each mouse (8 weeks of age) was injected with 5x10^6^ SEM cells and monitored for engraftment as described above. To determine whether dietary curcumin can enhance the activity of a conventional chemotherapeutic drug, all mice were injected i.p. with vincristine (0.5 mg/kg body weight) 3 times per week. The injection of vincristine began approximately 4.5 weeks after the injection of the leukemia cells and positive engraftment was well established. The total volume for each injection of vincristine was approximately 100 *μ*l and was adjusted weekly according to the body weight of each mouse. All animals were maintained on the control or curcumin diets during the chemotherapeutic treatment and the percentage of human leukemia cells was monitored in the mouse blood by flow cytometry as described above.

### Quantification of curcumin and curcumin metabolites

Blood from NOD/SCID mice was collected at the end of the experimental period from survivors and serum was prepared and frozen until use. Serum samples were thawed on ice and separated into 25 *μ*l aliquots for mock, glucuronidase and sulfatase digestions. A total of 5 *μ*l of a 0.2 mg/ml ethylenediaminetetraacetic acid and butylated hydroxytoluene was added to each aliquot prior to the addition of dimethoxycurcumin at 2 *μ*mol/l (recovery surrogate) and 500 nmol/l each of nitrophenyl glucuronide and nitrophenyl sulfate (digestion surrogates). As further recovery and digestion controls, 3 aliquots of normal mouse serum were spiked with curcumin at 200 nmol/l and the above surrogates. Digestions were performed with either 5 *μ*l 0.1 mmol/l ammonium formate pH 6.9 (mock digest), 5 *μ*l (0.5 kU) β-glucuronidase reconstituted in formate buffer, or 10 *μ*l sulfatase (0.11 U), and incubated at 37°C in the dark for 1 h in a shaking water bath. The serum digests were diluted with 75 *μ*l water and extracted twice with 400 *μ*l aliquots of cold ethyl acetate and once with 400 *μ*l cold acetonitrile. Each extraction step involved a 5-min 4°C vortex and a 10-min 4°C centrifugation at 14,000 x g. The organic phases were pooled in a screwcap polypropylene tube containing 5 *μ*l 50% methanolic glycerol and dried using a Savant SV110A SpeedVac (Savant Instruments Inc., Holbrook, NY). The dried extracts were reconstituted in 100 *μ*l of 100 nmol/l CUDA (internal standard prepared in methanol) by vortexing and then filtered through 0.1 *μ*m Amicon Ultrafree-MC durapore PVDF filters (Millipore, Billerica, MA) for 4 min at 4,000 x g prior to transfer to a glass insert in 2-ml amber vials for liquid chromatography (LC)-MS/MSanalysis. To assess matrix-dependent effects on analyte ionization, normalization solutions were prepared in triplicate for each batch of samples by enriching extracts of mock-digested normal mouse serum with target analytes immediately before filtration. Matrix effects on ionization were assessed relative to triplicate normalization solutions prepared in methanol containing 100 nmol/l CUDA.

An Acquity ultra performance liquid chromatograph (UPLC) (Waters Corp., Milford, MA) with an Acquity BEH C_18_ column (2x150 mm, 1.7 *μ*m particle size) held at 50°C was used to separate the analytes. A multistep gradient was run at a flow rate of 0.25 ml/min from 90% acidified water (0.1% formic acid) to neat acetonitrile over 13.5 min. Samples were maintained at 10°C within the autosampler during each sample batch and 10 *μ*l injections were made in partial loop with needle overfill mode. Mass spectral analysis of the UPLC effluent was performed with an API 4000 QTrap tandem mass spectrometer (AB Sciex, Foster City, CA) run in multi-reaction monitoring (MRM) mode. Residues were ionized using negative electrospray ionization (ESI^−^) for the first 7.75 min and positive electrospray ionization (ESI^+^) for the remainder of the run. Ionization and source parameters for the QTrap were: heater and source temperatures, 500°C; curtain gas flow, 40 ml/min; Gas 1/Gas 2, 40 ml/min; collision gas, high; ionspray voltage, 4.5 kV; and entrance potential (EP)/collision cell exit potential (CXP), 10 V. The limit of detection of curcumin was determined from visually defined peaks with a signal-to-noise ratio >2 (23). The optimized analyte parameters used in this study are summarized in Table I.

The quantification of curcumin was accomplished with a 7 point standard curve ranging from 1 to 3,000 nmol/l of curcumin, nitrophenyl sulfate and nitrophenyl glucuronide in methanol containing 100 nmol/l CUDA and 2 *μ*mol/l dimethoxycurcumin. Analyst 1.4.2 (AB Sciex) was used to integrate peak areas. Analyte signals were measured as peak area ratios to the internal standard, CUDA, to correct for evaporation and injection volume variability. A 1/x weighted linear regression of the standard curve was used to calculate the observed serum curcumin level, which was corrected for losses during digestion and extraction and effects of the matrix on ionization by dividing by the fraction of the dimethoxycurcumin signal recovered from each sample. The matrix and methanol normalization solutions were compared to distinguish matrix effects from extraction losses of the analytes.

### Statistical analysis

GraphPad software (GraphPad Software, Inc., San Diego, CA) was used for statistical analyses. For the parenteral administration of curcumin, the event-free survival (EFS) was calculated beginning with the initiation of i.p. treatment. For the dietary supplementation experiment, the EFS was calculated beginning with the day of injection of the leukemia cells. An event was defined as overt clinical illness that required euthanasia and included evidence of >20% weight loss, severe weakness or lethargy, or inability to reach food or water. The EFS was displayed as Kaplan-Meier plots with differences calculated using the log-rank test. Two-way analysis of variance (ANOVA) was used to compare the percentage of CD19^+^ cells between the groups over time. Data are displayed as arithmetic means ± standard deviation. Differences were considered significant at α (P-value) <0.05.

## Results

### Intraperitoneal injections of curcumin

**Figure 1.**
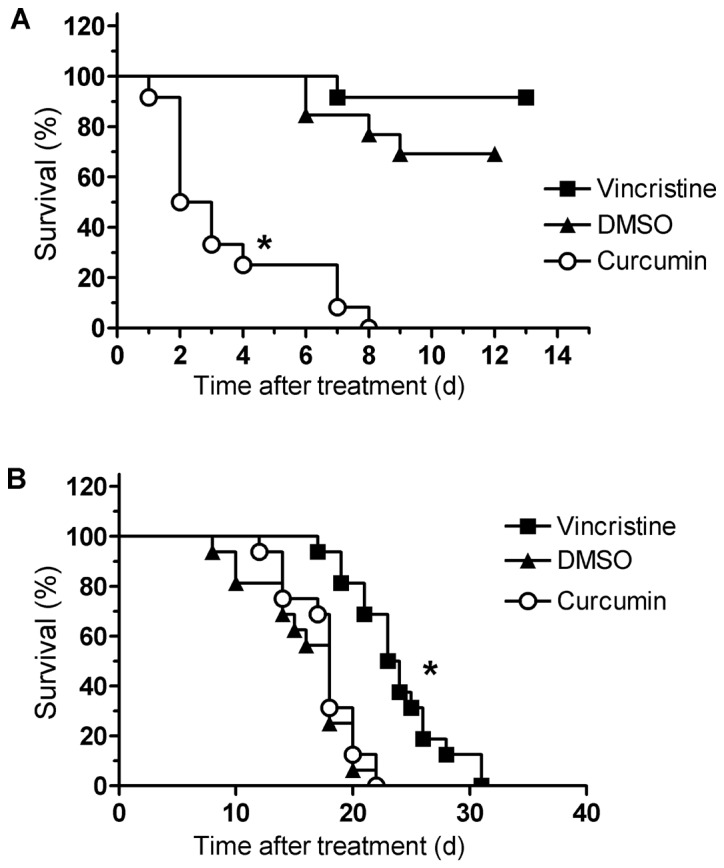
Intraperitoneally (i.p.) administered curcumin did not prolong survival in NOD/SCID mice engrafted with leukemia cells. (A) Mice were engrafted with the SEM cell line and treated i.p. with DMSO (n=13) or 100 mg curcumin/kg body weight (n=14) every other day, or 0.5 mg vincristine/kg body weight (n=13) once per week. A significant reduction in survival was observed for the curcumin-treated mice compared to the DMSO control and vincristine-treated groups (^*^P<0.0001). (B) Mice were engrafted with SEM cells and treated i.p. with DMSO or curcumin (5 mg/kg body weight) every other day, or vincristine (0.5 mg/kg body weight) 3 times per week for 4 weeks (n=16 per treatment group). Mice were euthanized when they showed >20% weight loss, lethargy, weakness, or inability to reach food or water. The vincristine-treated group showed an increased survival compared to both the DMSO- and curcumin-treatment groups (^*^P<0.0001). No differences in survival were observed between the DMSO- and curcumin-treated mice (P=0.41). The log-rank test was used to determine differences in survival between the different groups of mice. The survival data for the control and vincristine groups were used previously in a parallel analysis of resveratrol treatment (19).

A 100% engraftment of leukemia cells was observed in the mice receiving i.p. treatments of the vehicle (DMSO), curcumin, or vincristine. The 100 mg/kg i.p. dose of curcumin used initially in this study was toxic to the mice and reduced survival compared to the control group (Fig. 1A). Therefore, a dose-response analysis was performed to determine an appropriate dose for these mice. Doses of 5, 10, 25 and 50 mg/kg body weight were tested (n=4 mice per group). The 25- and 50-mg/kg i.p. dose showed toxicity and the presence of a curcumin precipitate in the abdominal cavity. The i.p. dose of 5 mg/kg body weight was chosen for the chemotherapeutic experiment as it showed no signs of toxicity and it was absorbed well with no signs of abdominal precipitation. At 5 mg/kg body weight, curcumin did not inhibit the growth of the engrafted leukemia cells compared to the control group, whereas vincristine treatment increased the survival of the mice (Fig. 1B). The body weights of the mice did not differ between the 2 groups (data not shown). The survival data for the control and vincristine groups in Fig. 1 were used previously in a parallel analysis of resveratrol treatment (19).

### Dietary curcumin

**Figure 2.**
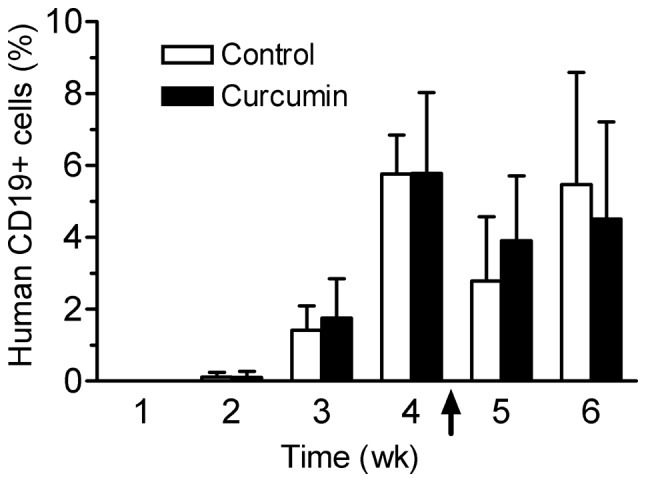
The percentage of human CD19^+^ cells in the mouse peripheral blood leukocyte (PBL) population was similar over time between the mice receiving control or curcumin diets. Mice (n=16 per group) were fed the control diet or a diet containing 0.5% w/w curcumin beginning at the age of 5 weeks. The mice were injected with SEM cells through the tail vein at 8 weeks of age. Two weeks after injection of leukemia cells, the PBLs were isolated from each mouse weekly and stained with PE-Cy7-conjugated anti-human CD19 and APC-Cy7-conjugated anti-mouse CD45 antibodies to monitor engraftment and growth by flow cytometry. Engraftment was monitored weekly. Three of the mice in the dietary curcumin group were not engrafted and were eliminated from the analysis. The arrow indicates the beginning of vincristine treatment. No difference in the percentage of CD19^+^ cells was observed between the 2 dietary groups. Data represent the means ± SD. The data for the control group was used previously in a parallel analysis of dietary resveratrol treatments (24).

**Figure 3.**
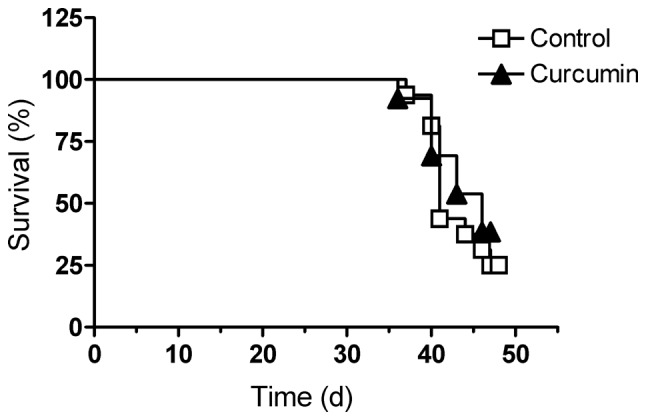
Dietary curcumin did not increase the survival of NOD/SCID mice with t(4;11) leukemia. The mice were fed the control diet or a diet supplemented with 0.5% curcumin w/w 3 weeks prior to the injection of the SEM leukemia cells (n=16 per dietary group). Vincristine treatment was commenced approximately 28–30 days after the injection of the leukemia cells. Mice were euthanized when they showed >20% weight loss, lethargy, weakness, or inability to reach food or water. Three of the mice in the dietary curcumin group were not engrafted and were eliminated from the analysis. The log-rank test was used to determine differences in survival between the dietary groups. No differences in survival were observed between the control and curcumin fed mice (P= 0.53). The data for the control group was used previously in a parallel analysis of dietary resveratrol treatments (24).

Dietary curcumin was evaluated for its potential to delay engraftment and sensitize leukemia cells to the chemotherapeutic agent, vincristine. All 16 mice in the control group were engrafted with leukemia cells. A total of 3 of the 16 mice in the group fed curcumin were not engrafted with the leukemia cells due to an inefficient tail vein injection of cells and these 3 mice were eliminated from the analyses. The mice were fed the intervention diets beginning at 5 weeks of age and the mice were injected with leukemia cells at the age of 8 weeks. There was no delay in engraftment of the leukemia cells in the mice fed curcumin compared to the mice fed the control diet (Fig. 2). Both groups showed similar percentages of human CD19^+^ leukemia cells at each weekly time-point. The mice fed the control and curcumin diets were injected i.p. with vincristine after engraftment was established to determine whether dietary curcumin enhances the efficacy of this chemotherapeutic agent. The arrow in Fig. 2 indicates the beginning of vincristine treatment. Although there was a drop in the percentage of human CD19^+^ leukemia cells in both groups of mice in response to vincristine, Fig. 2 shows that dietary curcumin did not enhance the vincristine-mediated reduction of leukemia cell burden. Both the control group and the curcumin-fed groups showed similar concentrations of human CD19^+^ leukemia cells in the mouse PBL population. The survival curve for the mice fed curcumin and control chow is shown in Fig. 3. Survival rates were similar in the 2 dietary groups. Body weights did not differ between the 2 groups (data not shown). The data for the control group in Figs. 2 and 3 were used previously in a parallel analysis of dietary resveratrol treatments (24).

### Bioavailability of curcumin

**Figure 4.**
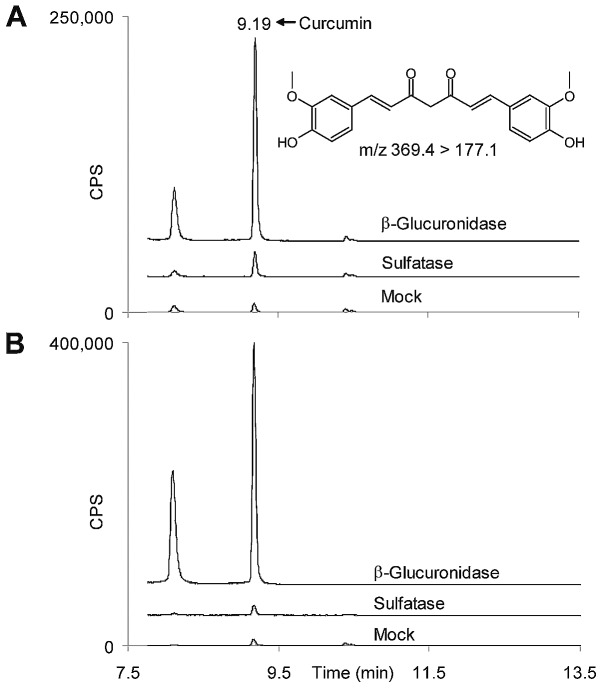
Liquid chromatography (LC)-tandem mass spectrometry (MS) profiles of curcumin from mouse serum after deconjugation of metabolites. (A) Serum samples were collected from 5 surviving mice 1 h after i.p. injection with curcumin and digested with buffer (mock), β-glucuronidase, or sulfatase. The profile shows the representative results from 1 mouse. Ultra-performance LC (UPLC)-MS/MS chromatograms show increased peak areas for curcumin after deconjugation with β-glucuronidase or sulfatase compared to the mock digestion, most notably with the glucuronidase digestion. (B) Serum samples were collected from 5 surviving mice that received diet supplemented with 0.5% w/w curcumin and digested with buffer (mock), β-glucuronidase, or sulfatase. The profile shows the representative results from 1 mouse. UPLC-MS/MSchromatograms show increased peak areas for curcumin after deconjugation with β-glucuronidase or sulfatase compared to the mock digestion.

**Figure 5.**
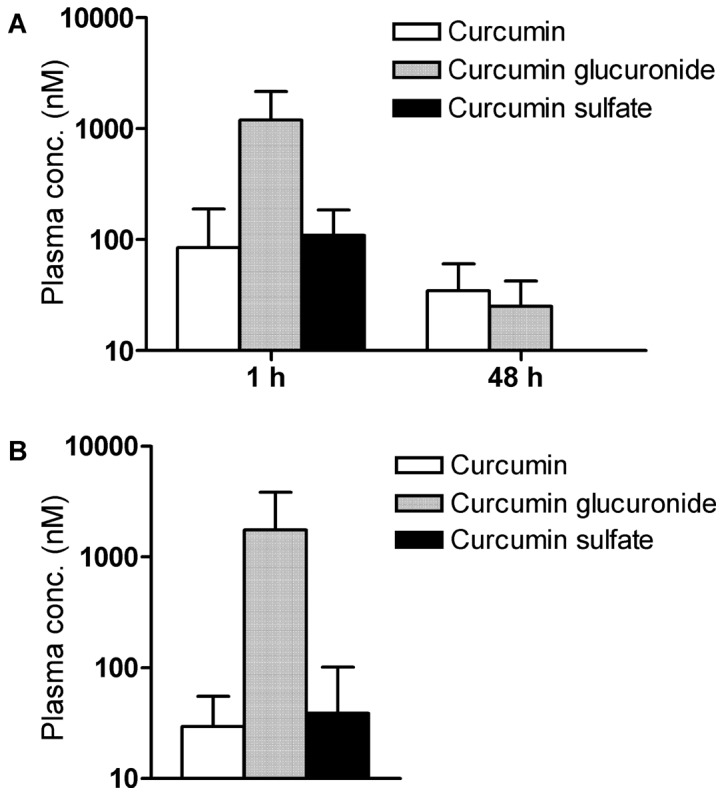
Curcumin glucuronide is the major form of curcumin in leukemic mice. (A) Mice were intraperitoneally (i.p.) injected with curcumin and the sera were collected at 1 and 48 h post-injection (n=5 for each time-point). The serum samples were analyzed by ultra-performance liquid chromatography (UPLC)-mass spectometry (MS)/MS after digestion with buffer (mock), glucuronidase and sulfatase. The concentrations of curcumin and its metabolites were estimated from the areas under the curves of the resulting curcumin peaks after the digestion. (B) Serum samples were collected from mice fed chow supplemented with 0.5% curcumin (n=5) and digested with buffer (mock), glucuronidase and sulfatase. The samples were analyzed as above and the concentrations of curcumin and its metabolites were estimated from the areas under the curves of the curcumin peaks after digestion. Data represent the means ± SD.

The bioavailability of curcumin after i.p. injection and dietary intervention were determined in a subset of surviving mice using tandem MS. The limit of detection for curcumin was estimated at 0.8 nmol/l in serum. Fig. 4 shows representative profiles of curcumin peaks after mock, glucuronidase and sulfatase digestions of mouse serum from the i.p. and dietary interventions, respectively. The deconjugation efficiency of the glucuronidase and sulfatase was >99% for all samples, as determined by the digestion surrogates, 4-nitrophenyl β-D-glucuronide and 4-nitrophenyl sulfate. Glucuronidase and sulfatase treatments resulted in increased peak intensities for curcumin in both profiles. The largest change in peak intensity for curcumin was observed after glucuronidase digestion, indicating that the majority of the circulating curcumin was in the form of glucuronide metabolites. Estimations of the amounts of curcumin aglycone and its metabolites were performed by measuring areas under the peak curves. Fig. 5 shows the approximate concentrations of circulating aglycone, glucuronidated and sulfated metabolites in the sera after i.p. injection (Fig. 5A) and dietary supplementation (Fig. 5B). For the i.p. injections, serum was prepared from mice 1 and 48 h post-injection (Fig. 5A) and show that curcumin and its glucuronidated metabolites, although still present at low nmol/l concentrations, were substantially reduced after 48 h. Serum samples were prepared from the euthanized mice at approximately the same time in the morning (roughly 10:00 am) for both the i.p. and feeding experiments. The mean concentration of curcumin glucuronide in the sera reached approximately 1.2 *μ*mol/l at 1 h post-i.p. injection and 1.7 *μ*mol/l for the dietary intervention.

## Discussion

Both orally and i.p. administered curcumin were evaluated as effective modes of preventing the growth of t(4;11) ALL cells in the NOD/SCID mouse leukemia model. Human feeding studies have shown that dietary curcumin up to 10 g is well tolerated, with minimal side-effects noted, such as mild diarrhea and headaches (25–27). In the current study, the estimated daily dose of curcumin in the mouse diet was 750 mg/kg body weight, which is equivalent to approximately 52 g of curcumin for a 70-kg human per day, based on body weight. No adverse effects were observed in the leukemic mice fed this amount of curcumin in their diet. However, this level of dietary curcumin did not decrease the engraftment and growth of leukemia cells in these animals. It should be noted that 3 mice in the dietary curcumin group were not engrafted with leukemia cells and were not included in the analysis of the dietary intervention. Although 100% engraftment was achieved in the other experimental groups described in this study, the lack of engraftment of these 3 mice was likely due to error in the tail vein injections and not to the dietary curcumin. This is supported by the data from the other 13 mice in the curcumin group who displayed the typical rapid growth of the SEM leukemia cells with no differences in survival or percentage of CD19^+^ cells compared to the control group.

While an i.p. dose of curcumin at 100 mg/kg body weight has been administered to rats in previous studies (22), we observed this amount of curcumin to be toxic to NOD/SCID mice engrafted with the t(4;11) ALL cell line, SEM. This observation resulted in a substantial reduction of the i.p. dose of curcumin that we subsequently administered to the mice in our study. Similarly, the NOD/SCID mice engrafted with the leukemia cells in our previous study were unable to tolerate high doses of i.p. injected resveratrol (19). For the i.p. curcumin administration, we observed the presence of an orange precipitate in the abdominal cavity at doses of 25 and 50 mg curcumin/kg body weight, indicating that curcumin was not absorbed efficiently at high concentrations in these mice. Other investigators have used a dose of 40 mg curcumin/kg body weight in Balb/c mice to treat ALL with an injection volume of 200 *μ*l (15). These investigators did not report toxicity or abdominal precipitates in their mice. However, the NOD/SCID mice were sensitive to DMSO at injection volumes of 100 *μ*l or more, showing temporary hindlimb paralysis (19). To alleviate the neurological sensitivity, we reduced the injection volume of DMSO to 40–60 *μ*l. A different vehicle that was more tolerable to the NOD/SCID mice may have allowed a greater injection volume and administration of higher concentrations of curcumin than reported in this study. At the i.p. dose of 5 mg curcumin/kg body weight, curcumin did not show any evidence of growth inhibition of t(4;11) ALL cells.

Pharmacokinetic studies have been performed using animals and humans to assess the bioavailability of curcumin and its metabolites following absorption. The tissue distribution and excretion of ^3^H-labelled curcumin has been evaluated in rats (28). While the majority of dietary curcumin is eliminated in the feces, 12 days after a single oral dose of 400 mg curcumin/kg body weight, approximately 13% of the radioactivity remained in the blood, 5% in the liver and 0.45% in the kidneys. In mice, a single i.p. dose of 100 mg curcumin/kg body weight resulted in approximately 177, 26, 27 and 8 *μ*g of curcumin/g of tissue in the intestines, spleen, liver and kidneys, respectively, 1 h post-injection (4). The total amount of curcumin per organ was calculated as 319.52 *μ*g in the intestine, 2.61 *μ*g in the spleen, 33.09 *μ*g in the liver and 3 *μ*g in the kidneys. The evaluation of the metabolites of curcumin from the plasma revealed the presence of glucuronide conjugates in this study. In humans, blood levels have been reported to peak at approximately 1.8 *μ*mol/l in the serum of individuals 2 h after consuming 8 g curcumin (29). Other studies have detected both glucuronidated and sulfated forms of curcumin in the plasma of humans after oral administration (25). Vareed *et al*(30) detected a maximum of approximately 2 mg/l of curcumin glucuronide and 1 mg/l curcumin sulfate in human plasma after consumption of a single dose of 10 g curcumin. These authors found that the glucuronidated and sulfated metabolites were almost exclusively present in the plasma with little or no detectable curcumin aglycone. In the current study, curcumin glucuronide and sulfate were the major curcumin metabolites found in mice after i.p. and dietary treatments, which concurs with the data from the studies mentioned above.

Curcumin has been reported to exert cancer-preventive activities, most notably in animal studies of epithelial-derived cancers, such as colon, skin and stomach cancers (6–8), where the unmetabolized aglycone of this molecule would be more accessible to cancerous epithelial cells and present at its highest level. Importantly, the majority of recent clinical trials using curcumin have focused on oral and gastrointestinal cancers (3). A Phase IIa clinical trial for the prevention of colorectal neoplasia by oral curcumin was performed using 2- and 4-g doses of curcumin in 41 subjects with aberrant crypt foci (31). The authors reported that curcumin at an oral dose of 4 g/day for 30 days produced a 40% reduction in aberrant crypt foci. In another human study, dietary curcumin significantly reduced the relapse of ulcerative colitis, a perceived precursor to colon cancer, during a 6-month co-treatment with sulfasalazine or mesalamine (32). Curcumin has been evaluated as a potential treatment for pancreatic cancer in humans with limited results (33). A total of 2 out of 21 evaluable patients had a biological response to 8 g/day of orally administered curcumin. These 2 patients displayed stabilization of disease or marked, but brief, tumor regression. The authors attributed the lack of responsiveness to the low bioavailability of curcumin.

There has been considerable interest in developing novel delivery systems to increase the bioavailability of absorbed curcumin. Delivery systems include nanoparticles, nanogels, liposomes and nanocrystals (reviewed in ref. 34). Poly(lacticco-glycolic acid) nanoparticles of curcumin showed a 5.6-fold greater bioavailability and longer half-life than native curcumin in rats (35). Nanoemulsions of curcumin with polyethylene glycol and the castor oil derivative, Cremaphor EL^®^, increased the maximum concentration of curcumin 40-fold in mice (36). Curcumin has been reported to chemosensitize a number of different cancers to the activities of standard chemotherapeutic drugs (37). Liposomally encapsulated curcumin at a concentration of 25 mg/kg body weight synergized with paclitaxel to reduce cervical tumor incidence and volume in mice (38). Oil-in-water nanoemulsions of curcumin combined with paclitaxel have been shown to significantly reduce tumor volume in mice bearing subcutaneous ovarian adenocarcinoma by delaying the proliferation and growth of the tumor cells (39).

At the concentrations described in this study, curcumin, whether injected or fed, failed to reduce the growth of the SEM leukemia cells in the NOD/SCID mouse model. The SEM leukemia line was derived from a patient with relapsed t(4;11) ALL, indicating that these cells had gone through chemotherapeutic selection processes resulting in chemotherapy resistance. We have shown that these SEM leukemia cells underwent a rapid reversion to a chemotherapy-resistant phenotype in NOD/SCID mice when challenged with vincristine, which is a standard chemotherapeutic agent used in the treatment of this disease (40,41). It is still a possibility that curcumin may be effective against t(4;11) ALL at the time of diagnosis before resistance mechanisms are enhanced and the analysis of the efficacy of curcumin against t(4;11) ALL needs to be further examined. Curcumin may also prove to be effective against less aggressive forms of ALL. However, further evaluation of this agent should take into account the rapid metabolic transformation of curcumin that occurs *in vivo* and the generation of a unique delivery system plus the use of a combinatorial chemotherapeutic approach will likely be required for the effective treatment of this disease.

## Figures and Tables

**Table I.** Parameters for tandem mass spectrometric analysis of curcumin in mouse serum.^a^

Analyte (in order of elution)	Precursor (m/z)	Product (m/z)	+ DCP (V)	+ CE (V)	Retention time (min)	Spiked normal serum (*μ*M)
4-Nitrophenyl β-D-glucuronide	314.2	138.2	−50	−30	5.07	0.5
4-Nitrophenyl sulfate	218.2	138.2	−50	−30	5.71	0.5
Curcumin	369.4	177.1	+50	+30	9.19	0.2
CUDA	341.4	216.2	+58	+25	9.62	n/a
Dimethoxycurcumin	397.4	313.1	+25	+20	10.31	2.0

a API 4000 QTrap MS/MS parameters: ionspray voltage, 4.5 kV; curtain gas flow, 40 ml/min; collision gas, high; heater and source temperatures, 500°C; EP/CXP, 10 V; Gas 1/Gas 2, 40 ml/min. DCP, direct current plasma; CE, capillary electrophoresis.
